# Reducing Burnout and Promoting Health and Wellness Among Medical Students, Residents, and Physicians in Alberta: Protocol for a Cross-Sectional Questionnaire Study

**DOI:** 10.2196/16285

**Published:** 2020-04-17

**Authors:** Esther Kim, Robert Mallett, Marianne Hrabok, Yajing Alicia Yang, Chantal Moreau, Izu Nwachukwu, Maryana Kravtsenyuk, Adam Abba-Aji, Daniel Li, Vincent I O Agyapong

**Affiliations:** 1 Department of Psychiatry Faculty of Medicine and Dentistry University of Alberta Edmonton, AB Canada; 2 Department of Psychiatry Cumming School of Medicine University of Calgary Calgary, AB Canada; 3 Alberta Health Services Edmonton, AB Canada

**Keywords:** burnout, psychological, wellness, resilience, burnout measures, burnout interventions, delivery of health care, medicine, work life balance

## Abstract

**Background:**

Burnout is an increasingly common and insidious phenomenon experienced by workers in many different fields, although it is of particular concern among physicians and trainees due to the nature of their work. It is estimated that one-third of practicing physicians will experience burnout during their career, and this rate is expected to continue to increase. Burnout has significant implications, as it has been identified as a contributor to increased medical errors, decreased patient satisfaction, substance use, workforce attrition, and suicide.

**Objective:**

This study will evaluate the prevalence and impact of burnout on physicians, residents, and medical students in Alberta.

**Methods:**

Quantitative and qualitative data collected through self-administered, anonymous, online questionnaires will be used in this cross-sectional provincial study design. Data collection tools were developed based on published literature and questions from previously validated instruments. The tools capture relevant demographic information, mental health status, and rates of burnout, as well as factors contributing to both burnout and resilience among respondents. We anticipate a sample size of 777 medical students, 959 residents, and 1961 physicians to represent the respective ratios of trainees and practicing physicians in the province of Alberta.

**Results:**

Study recruitment will begin in September 2020, with 4 weeks of data collection. The results of this study are anticipated within 12 months from the end of data collection. It is expected that the results will provide an overview of the prevalence of burnout among those training and working in medicine in Alberta, identify contributors to burnout, and help develop interventions aimed at reducing burnout.

**Conclusions:**

This study’s aim is to examine burnout prevalence and contributing factors among medical trainees and physicians in Alberta. It is expected that the results will identify and examine individual and organizational practices that contribute to burnout and help develop strategies and interventions focused on mitigating burnout and its sequelae.

**International Registered Report Identifier (IRRID):**

PRR1-10.2196/16285

## Introduction

Burnout is a complex phenomenon that is characterized by three domains: emotional exhaustion, depersonalization, and reduced personal accomplishment [1]. Emotional exhaustion refers to excessive emotional fatigue from overwhelming demands in the workplace; depersonalization refers to the impersonal feelings or indifference experienced towards patients; and reduced personal accomplishment refers to deficits in one’s conviction that they are competent and successful in their work [1]. The term burnout was coined in the 1960s [2] to describe the psychological and emotional distress experienced by health care staff working at a clinic for the underprivileged. Today, the term reflects an individual’s negative response to the demands of the workplace in the setting of chronic affective and interpersonal stressors [3-6]. In the medical profession, it is felt that chronic exposure to stress is the primary cause of emotional exhaustion, while depersonalization occurs as physicians begin to treat patients indifferently and develop some degree of pessimism towards their colleagues and profession. A lack of personal achievement is thought to occur when practitioners withdraw from their responsibilities at work [7]. This study protocol aims to provide an overview of the prevalence of burnout among medical trainees and physicians within Alberta and to identify systemic and personal factors contributing to burnout in order to develop potentially mitigating interventions.

Among the general working population, the prevalence of burnout is suggested to range from 7% to 25%, with discrepancy among rates secondary to the use of different scales and cutoff levels in population-based studies [8-11]. The majority of burnout studies have focused on specific subgroups within the context of human services occupations, including but not limited to education, health care, and support services. While burnout is not exclusive to physicians, the profession’s increasing workload, challenging work environment, increasing documentation demands (including the emergence of electronic health records), threats of litigation, and challenges to work-life balance render physicians particularly vulnerable to burnout [12]. Burnout affects physicians at all stages of their careers. Research suggests that burnout takes root during medical school and continues into residency and practice [13]. Romani and Ashkar [7] reported that up to 50% of medical students, 76% of surgical and internal medicine residents, and 45% of practicing physicians in the United States reported at least one symptom of burnout. These frequencies, when compared to the general working population, have raised concerns about burnout and its effects on the medical profession.

Medical students with high burnout scores were more likely to engage in unprofessional behaviors, such as cheating and plagiarism, which may undermine future professionalism (eg, managing conflicts of interest, reporting incompetent colleagues, adhering to appropriate prescription practices) [14]. Additional research has suggested that higher burnout scores are associated with lower empathy scores, less altruistic views of medicine as a career, and consideration of leaving the medical profession altogether [13,15-17]. A national study of medical students in the United States reported that increased risk of alcohol abuse was independently associated with burnout [18]. Burnout in students was also a predictor of suicidal ideation, which is particularly worrisome when considered in combination with alcohol abuse [19]. Dyrbye et al [13] found that depression and suicidal ideation were most pronounced during medical school and diminished as individuals’ careers progressed through training into practice.

In first-year internal medicine residents, higher burnout scores were associated with lower reported quality of life and education with more negative patient-doctor relationships, negative interactions with other health care professionals, and perceived increase in medical errors with poorer patient safety [20,21]. Burnout, depersonalization, and fatigue were highest during residency, with fatigue being an independent factor contributing to errors, injuries, and motor vehicle collisions [13]. In a US national study involving internal medicine residents, higher burnout scores, specifically high emotional exhaustion, were associated with lower scores on a standardized national exam [22]. Suicidal thoughts were also more prevalent in residents with burnout than those without [23]. It is suggested that the likelihood of burnout is highest within the first year of residency but can persist throughout the duration of residency [24].

Overall, one-third of all practicing physicians are expected to experience burnout during their career, and the rates of burnout appear to be worsening over time [25]. It appears that rates of burnout among practicing physicians are relatively consistent across geographical regions, with one-third of physicians in Yemen, Qatar, and Saudi Arabia reporting burnout, similar to self-reported rates in the United States [7]. Burnout among physicians increases medical errors, negatively impacts patient safety, and leads to lower patient satisfaction [2,26]. Alcohol abuse was independently associated with burnout among US physicians and surgeons [27,28]. Grinspoon [12] questioned the role of burnout in the roughly 400 physician suicides that occur each year in the United States and noted the increasing numbers of physicians leaving the profession mid-career.

The phenomenon of burnout has yet to be defined by specific criteria. Until now, burnout has typically been measured using the Maslach Burnout Inventory, a 22-question, self-reported, validated survey that assesses the key domains of emotional exhaustion, depersonalization, and personal accomplishment on a 7-point Likert scale [1]. Alternative scales have also been developed and used in physician and medical trainee populations to further characterize the heterogeneity of burnout [29-33]. For example, the Oldenburg Burnout Inventory and Utrechtse Burnout Scale distinguish burnout domains in the context of job demands and job resources [33,34]. The Copenhagen Burnout Inventory describes burnout domains in relation to personal, work, and client-related factors [35]. These alternative scales reflect the multifaceted interplay of diverse factors that contribute to burnout among physicians and medical trainees [29-33].

Using various scales in qualitative and quantitative studies, evidence has shown that burnout is associated with a variety of individual, occupational, and organizational factors. Based on the 5-dimensional model of personality coined by Goldberg in 1990, neuroticism has been shown to potentially predispose individuals to developing burnout through maladaptive coping mechanisms [36]. Longitudinal data suggest associations exist between neuroticism and emotional exhaustion among physicians and that these physicians report less satisfaction with medicine as a career [37]. Psychosocial stressors outside of medicine and the workplace such as illness, family-related conflict, or financial concerns can also increase trainees’ vulnerability to burnout [24,38,39]. For instance, residents with significant amounts of educational debt were more likely to have higher rates of burnout [22].

Burnout rates are not only affected by work-related factors such as area of practice, hours worked per week, lower autonomy, and number of call shifts per week but also non work-related factors such as age, gender, and number of children [7,40,41]. Among Hungarian general practitioners and residents, younger age, male gender, fewer years of experience, and increased number of dependents such as children were correlated with higher rates of burnout [40]. Such results were postulated to be secondary to less work experience leading to increased stress in the workplace as well as difficulty with balancing the demands of home and work life, leading to higher levels of emotional exhaustion and perpetuating depersonalization [40].

Unfortunately, there is a dearth of data regarding interventions that have proven beneficial in reducing burnout rates, including stress management courses, mindfulness, brief exercise, and short-term counselling. In interventions that have been implemented, there is insufficient evidence to suggest that they have a meaningful impact past the intervention period [7]. Furthermore, interventions aimed at reducing burnout must cater specifically to each stage of practice; it is unlikely that a single solution will be uniformly effective for medical school training, residency, and independent practice [7].

Within Canada, physician health and wellness initiatives have been evolving, with the development of professional support networks, wellness curriculums, adoption of health as a component of core CanMEDS competencies, and risk-management strategies adapted from organizational and occupational systems [42,43]. However, despite targeted strategies, such as mindfulness-based therapy, resilience training, and access to professional services, the 2018 Canadian Medical Association National Physician Health Survey suggests ongoing high rates of burnout (30%) and positive screening for depression (34%) among physicians and residents [44]. Additionally, there are insufficient data regarding burnout rates within each province in Canada. This poses further questions because health care is delivered on a provincial level and, as such, can vary in terms of policies from province to province.

The goal of this proposed study is to evaluate the impact of burnout on physicians, residents, and medical students in Alberta and identify the individual, occupational, and organizational variables that influence burnout dimensions. The results of this study will be used to propose specific interventions and preventative courses of action to mitigate burnout and subsequent impairment among physicians, residents, and medical students in Alberta. Given this overall goal of our study, our specific research questions include:

- What are the prevalence rates and correlates of burnout among medical students, residents, and physicians in Alberta?
- What are the perceptions and experience of respondents about the consequences of burnout on the personal and professional life of medical students, residents, and physicians in Alberta?
- What interventions can be implemented to mitigate burnout and promote health and wellness among medical students, residents and physicians in Alberta?

It is hypothesized that the findings will confirm that the prevalence rates of burnout among trainees and physicians are consistent with those reported in other jurisdictions. Our study will also identify whether personal and organization-related factors contribute to burnout among respondents, and lastly, respondents will identify multiple interventions that can be implemented to promote health and wellness among medical students, residents, and physicians in Alberta.

## Methods

### Study Setting

This study will be conducted in Alberta, a western province of Canada with a population of 4,286,134 in 2017 [45]. Alberta is divided into 5 administrative health regions, namely the Edmonton, Calgary, Southern, Northern, and Central zones. Health care is administered mainly through Alberta Health Services and Primary Care Networks and Medicentre Clinics. As of December 2018, 10,674 physicians were listed on the in-province registers held by the College of Physicians and Surgeons of Alberta [46]. There are two medical schools in the province that train both medical students and residents: University of Alberta, Faculty of Medicine and Dentistry and University of Calgary, Cumming School of Medicine. As of December 2018, there were 1594 postgraduate medical residents and 1148 medical students registered with the College of Physicians and Surgeons of Alberta [46].

### Study Design, Sample Size, and Institutional Review Board Approval

Quantitative and qualitative data collected through self-administered, anonymous, online questionnaires will be used in this cross-sectional provincial study design. The study will be comprised of 3 arms including medical students, residents, and practicing physicians within Alberta. Each arm will have a specific survey oriented towards data collection that is relevant to the study population in question.

For the medical student arm of the study, given the total medical student population in Alberta of 1148 [46], an anticipated sample size of 777 was determined based upon a 95% confidence level and a margin of error of 2% for prevalence rate estimates for medical student burnout. Similarly, for the resident arm of the study, given the total resident population in Alberta of 1594 [46], an anticipated sample size of 959 was determined based upon a 95% confidence level and a margin of error of 2% for prevalence rate estimates for resident burnout. Finally, for the physician arm of the study, given the total physician population in Alberta of 10,674 [46], an anticipated sample size of 1961 was determined based upon a 95% confidence level and a margin of error of 2% for prevalence rate estimates for physician burnout.

This study will be carried out in accordance with the recommendations of the Health Ethics Research Board of the University of Alberta and the Conjoint Health Research Ethics Board of the University of Calgary. The study will also be conducted in accordance with the Declaration of Helsinki (Hong Kong Amendment) and Good Clinical Practice (Canadian Guidelines). All participants will be provided with an online information leaflet and will provide informed consent prior to participation, in accordance with the Declaration of Helsinki. The protocol has received ethical approval by the Health Ethics Research Board of the University of Alberta (reference number Pro00091436) and the Conjoint Health Research Ethics Board of the University of Calgary (REB19-1457).

### Data Collection Tools

Data collection tools for each arm of the study were developed based on published literature and questions from previously validated instruments. The general constructs of interest included relevant demographic information, current practice and career planning, general health status, mental health status, and rates of burnout, as well as factors contributing to both burnout and resilience among respondents. The qualitative portion of the study will be gathered from conceptualized themes arising from the results within each of the constructs of interest and its association to burnout. In addition, each survey will include open-ended questions to facilitate qualitative data collection. Standardized measures from which questions were selected and included in the survey were the Maslach Burnout Inventory, Patient Health Questionnaire 9, Canadian Medical Association National Physician Health Survey, Mini Z burnout survey, Professional Fulfillment Index, and two-item Connor-Davidson Resilience Scale [1,44,47-51]. These standardized measures will provide information for the quantitative portion of the study results.

Prior to the finalization of the study survey, the research team sought feedback regarding the developed data collection tools and selected standardized instruments from stakeholders in Alberta, including representatives of the College of Physicians and Surgeons of Alberta’s Physician Wellness Program, Alberta Medical Association’s Physician and Family Support Program, University of Alberta Faculty of Medicine & Dentistry’s Offices of Advocacy & Wellbeing, and University of Calgary Department of Medicine’s Physician Wellness and Vitality Program. Feedback from these stakeholders was used to revise the data collection tools to be used in the study. The revised baseline data collection tools were then pretested on 5 randomly selected representatives from each arm of the study. Feedback from the pretest was further used to revise the data collection tool before it was finally adopted for use in the study.

### Eligibility, Data Collection Procedures, and Analysis

All medical students and residents at the University of Alberta and the University of Calgary as well as physicians registered with the College of Physicians and Surgeons of Alberta and practicing medicine in the province of Alberta are eligible to participate in the study. Data collection will occur online using “Survey Select,” an electronic survey platform, which is hosted by Alberta Health Services (the provincial health authority).

A link to the online information leaflet, consent page, and survey questionnaires will be emailed to all medical students and residents in Alberta through the mailing list of the Offices of the Undergraduate and Post Graduate Medical Education at the University of Alberta and University of Calgary. In order to reach physician respondents, the link will be sent through multiple sources, including the Alberta Medical Association, Alberta Health Services, Primary Care Networks, and Medicentre organizations. Data collection will occur over 4 weeks, and reminder emails will be sent to all eligible respondents weekly. Quantitative data will be analyzed using SPSS 20.0 (IBM Corp, Armonk, NY). Descriptive and inferential statistics will be used to describe demographic characteristics and study variables. Qualitative data will be analyzed using manual thematic analysis.

## Results

We anticipate that the study recruitment will begin in September 2020, with 4 weeks of data collection, and study findings will be available within 12 months following completion of data collection. It is expected that the findings will confirm that the prevalence rates of burnout among trainees and physicians are consistent with those reported in other jurisdictions. In addition, we expect to uncover specific contributors to burnout, which will serve as an opportunity for identification of meaningful solutions from the respondents’ perspectives.

We intend to disseminate the research findings at several levels, including trainees, physicians, academics, researchers, and health care organizations, as well as membership associations and licensing colleges. This information will be disseminated to academics and stakeholders through research forums and peer-reviewed journals. The expected findings will become available to trainees and physicians through the same communication channels used to provide the initial link for data collection purposes. Namely, this will include the Offices of the Undergraduate and Postgraduate Medical Education at the University of Alberta and University of Calgary, Alberta Medical Association, Alberta Health Services, Primary Care Networks, and Medicentre organizations.

## Discussion

This study will contribute to and build on current knowledge by identifying rates of burnout among stages of training and practice in the medical profession, uncovering specific contributors to burnout, and identifying potentially meaningful solutions from the respondents’ perspectives. This study is relatively unique for the following reasons: physicians at multiple stages of training and practice are included; both antecedents to burnout and possible interventions will be examined; and the study population will be broad but within the context of the local health system in Alberta.

It is estimated that 30% of medical trainees and physicians in Alberta will experience burnout based on national study data [44]. Poor work-life balance in the setting of increasingly challenging work environments and additional psychosocial stressors outside of medicine can make physicians and trainees particularly vulnerable to burnout [12,24,38,39]. Both work-related factors (eg, area of practice, hours worked per week, lower autonomy, and number of call shifts per week) and non-work related factors (eg, age, gender, number of dependents, and fewer years of experience) have been shown to affect burnout rates [7,40,41].

While the personal impact of burnout on physicians and medical trainees is substantial, the subsequent costs to patients and the health care system in general are most concerning. Trainees and physicians experiencing burnout are more likely to tolerate unprofessional behavior and encounter negative patient-physician relationships as well as negative interactions with other health care professionals, thereby considering leaving the medical profession mid-career [14,20-22]. Such erosion in practitioners’ confidence can subsequently place additional burdens on the health care system, with human resource shortages and expanded waiting times [26,52]. A Canadian study published in 2014 estimated that the total cost of burnout from physicians retiring prematurely or reducing clinical hours was approximately Can $213.1 million, revealing the financial extent and impact of the sequelae of burnout on the health system [26].

Unfortunately, there is a paucity of data regarding interventions that have proven beneficial in impacting burnout rates past the intervention period. Mentorship programs and good occupational leadership have been proposed to potentially influence the wellbeing and satisfaction of individual physicians working in health care organizations [53]. Nevertheless, interventions aimed at reducing burnout will need to be tailored to the stage of training and practice, which will require recognition of the factors contributing to burnout at each stage.

In summary, although physician health and wellness are steadily gaining recognition as a serious issue, gaps remain regarding how burnout affects trainees and physicians in Alberta, the consequences that derive from burnout, and the interventions used to reduce burnout. What appears to be needed is an understanding of the factors contributing to burnout in the context of local health care systems. Valuable insight can be gained from the perceptions and experiences of trainees and physicians in Alberta. These can in turn inform organizational strategies to mitigate burnout and promote wellness within the medical profession, thereby impacting patient care and reducing the burden on health care systems.
